# [Retracted] Treatment with a selenium-platinum compound induced T-cell acute lymphoblastic leukemia/lymphoma cells apoptosis through the mitochondrial signaling pathway

**DOI:** 10.3892/ol.2025.15303

**Published:** 2025-09-29

**Authors:** Feifei Wu, Wei Cao, Huaping Xu, Mingxia Zhu, Jing Wang, Xiaoyan Ke

Oncol Lett 13: 1702–1710, 2017; DOI: 10.3892/ol.2017.5666

Subsequently to the publication of the above paper, an interested reader drew to the authors’ attention that two of the flow cytometric (FCM) plots featured in Fig. 6C on p. 1708 [namely, the Molt-4/Control and Molt-4/25 μM+NAC (20 mM) plots] were unexpectedly similar, even though the reported gated percentages were different. Upon analyzing the data independently in the Editorial Office, it was also noted that, regarding the western blots featured in Fig. 7A on p. 1709, the protein bands shown for the Cleaved PARP experiment with the Jurkat cell line were strikingly similar to the data shown for the Bcl-2 experiment with the Molt-4 cell line, albeit the protein bands were included in the figure in different orientations.

Given that it has come to light that a couple of the figures in this paper were apparently misassembled, which might have had an adverse effect on the interpretation of the results and conclusions in the article, the Editor of *Oncology Letters* has decided that this paper should be retracted from the publication on account of a lack of confidence in the presented data. The authors were asked for an explanation to account for these concerns, but the Editorial Office did not receive a reply. The Editor apologizes to the readership for any inconvenience caused.

